# Extracellular protein degradation via the lysosome

**DOI:** 10.1038/s42004-020-00397-8

**Published:** 2020-10-30

**Authors:** Mohamed A. Eldeeb, Cornelia E. Zorca, Thomas Goiran

**Affiliations:** Department of Neurology and Neurosurgery, Montreal Neurological Institute, McGill University, Montreal, Quebec Canada

**Keywords:** Proteins, Lysosomes

## Abstract

Tight regulation of protein levels is so crucial for cellular function that mammalian cells have evolved two parallel degradation systems. This article discusses how these systems can be exploited to selectively target proteins of interest for therapeutic purposes.

The development of small molecules and biological tools to modulate diverse cellular processes is crucial for finding novel therapies to treat human diseases. Classical pharmacological therapies rely on targeting proteins with catalytic active sites and often involve large-scale screening of chemical libraries to identify small molecules that alter target protein activity. However, catalytic proteins, such as ion channels, nuclear receptors, and GPCRs, constitute only twenty percent of the cellular proteome that can be probed with small molecules^1^. This problem imposes the challenge of finding alternative strategies to target proteins that are not sensitive to pharmacological modulation. Advances in understanding the two major degradation systems in mammalian cells, the ubiquitin proteasome system (UPS) and the autophagic-lysosomal pathway (ALP), have paved the way for a new paradigm in molecular pharmacology and therapeutics. This paradigm exploits these endogenous cellular proteolysis pathways to directly deplete, rather than inhibit, target proteins.

## Intracellular protein degradation

**Fig. 1:**
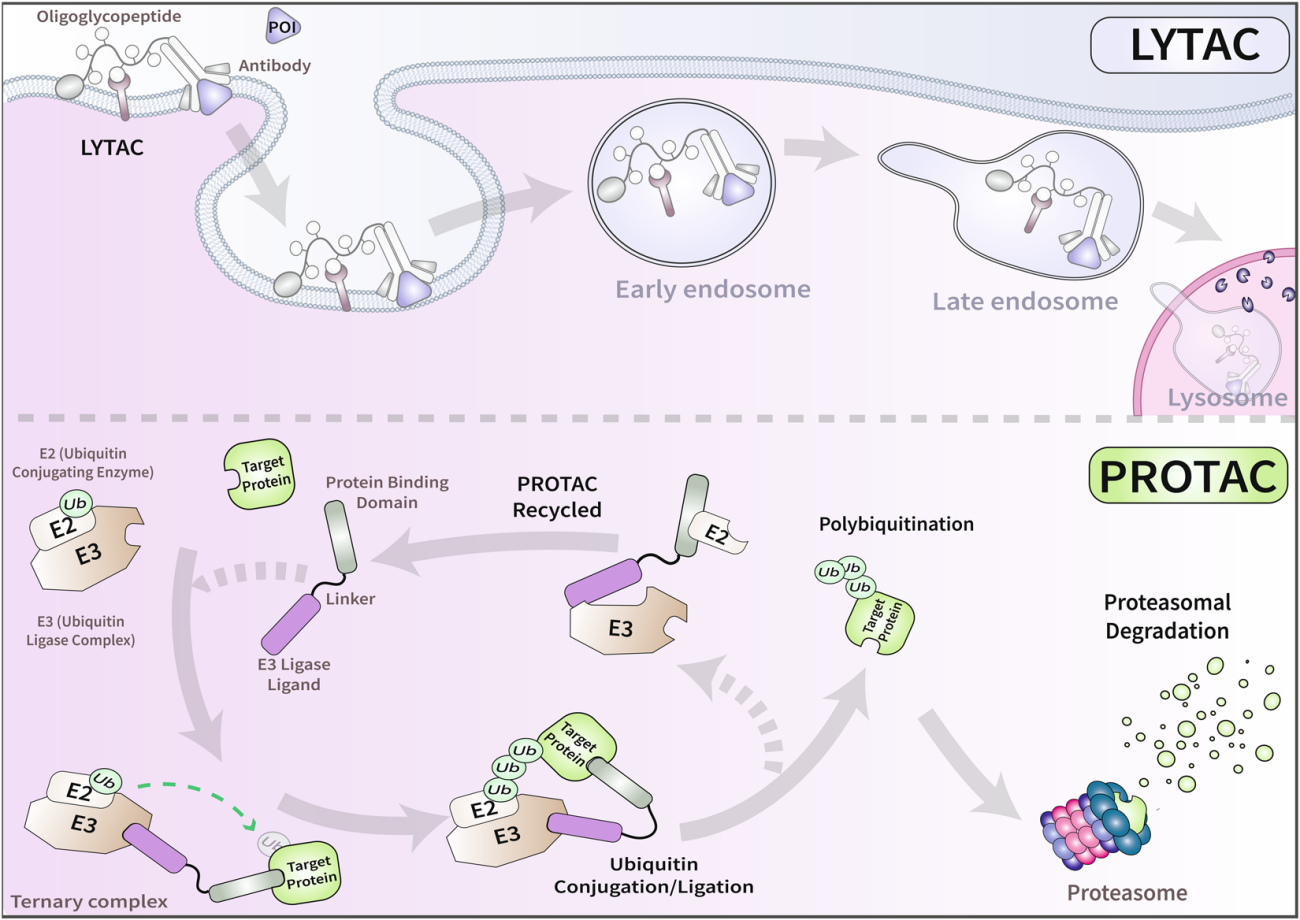
Comparison between LYTAC and PROTAC. In the case of LYTACs, the trimeric complex consists of the target extracellular protein, the chimera and a lysosome shuttling receptor expressed at the cell surface, which travels through the endo-lysosomal system for degradation (top panel). By contrast, in the case of PROTAC, the trimeric complex is composed of the target intracellular protein, the chimera and an E3 ligase, which targets the ubiquitinated cargo for destruction by the proteasome (bottom panel).

Protein degradation regulates numerous aspects of cellular homeostasis. In recent years, the endogenous protein degradation machinery has been reprogrammed to eliminate a variety of intracellular substrates through an approach named targeted protein degradation (TPD)^2^. One of the earliest reported TPD platforms, proteolysis targeting chimeras (PROTACs), have recently entered clinical trials^3^. PROTACs consist of two moieties, which bind a target and a ubiquitin ligase (E3), separated by a flexible linker^2^. The E3 ligase conjugates the target with ubiquitin, marking it for subsequent proteasomal degradation (Fig. 1, top panel). In a recent report, Qu et al.^4^ explored the use of PROTACs to specifically degrade α-synuclein (α-syn), the primary constituent of Parkinson’s disease-associated aggregates called Lewy bodies. Specifically, the authors demonstrated that either exogenous expression or direct introduction of α-syn PROTACs into cell lines or primary neurons had the remarkable ability to modulate α-syn protein levels in a proteasome-dependent manner^4^. Although PROTACs can, in principle, target any intracellular protein, extracellular and transmembrane proteins are inaccessible to this approach. Recent investigations by Banik et al.^5^ and Anh et al.^6^ have overcome this limitation by engaging the endo-lysosomal degradation pathway through the development of lysosome targeting chimeras (LYTACs).

## Extracellular protein degradation

Unlike PROTACs, LYTACs form a ternary complex that captures the extracellular target protein through a small molecule or antibody conjugated with a ligand for a co-opted lysosome shuttling receptor that localizes at the plasma membrane and directs cargoes for lysosomal degradation (Fig. 1, bottom panel). Specifically, the first LYTAC developed by Banik et al.^5^ harbored an oligoglycopeptide moiety (polyM6Pn) that functioned as a ligand for the cation-independent mannose-6-phosphate receptor (CI-M6PR) and efficiently induced the degradation of different classes of extracellular and transmembrane proteins^5^. Although LYATCs extend the reach of TPD strategies by employing the ALP instead of the UPS to degrade distinct classes of proteins, some features of this tool remain to be understood and improved. For instance, it is presently unknown whether acquired mutations in genes that affect the intracellular transport of M6PR, such as previously reported KIF13A mutations, can diminish the applicability of this technique^7^. In addition, the CI-M6PR receptor is essential for LYTAC-mediated target internalization and removal, so cell type variations in the expression levels of this ubiquitous receptor directly impact degradation efficiencies. Banik et al.^5^ indeed observed such variations in two of the cell lines examined. Perhaps, most importantly, the use of a ubiquitously expressed CI-M6PR receptor allows neither for spatial nor for temporal control of target degradation, a major limitation if administered systemically.

Addressing these points, Ahn et al.^6^ developed a novel type of LYTAC that utilizes the asialoglycoprotein receptor (ASGPR), a liver-specific lysosomal shuttling receptor, to direct the degradation of *N*-acetylgalactosamine (GalNac)-conjugated complexes. In an elegant series of proof-of-concept experiments, the authors demonstrated that treatment with a blocking antibody against EGFR linked to GalNac (Ctx-GalNac) led to a marked reduction in EGFR levels both in cell lines and in mice^6^. Notably, treatment with Ctx-GalNac LYTACs did not affect lysosomal function as evaluated by measurements of Cathepsin B activity, among other parameters, suggesting that these chimeric molecules do not overwhelm the organelle that degrades them. In addition, different Ctx-GalNac adducts exhibited distinct in vivo clearance rates, depending on the GalNac conjugation site within the antibody. Nonspecifically conjugated Ctx-GalNac LYTACs exhibited rapid clearance within 6 h, whereas site-specific conjugated species persisted for 3 days. Thus, the active degradation time window of Ctx-GalNac LYTACs can be modulated by design. Further questions concerning the potential of LYTACs to induce multiple rounds of target degradation similar to PROTACs, as well as the metabolic and pharmacokinetic properties of LYTACs under normal and disease conditions remain to be addressed in future studies.

## Outlook

In summary, PROTACs and LYTACs represent powerful and complementary therapeutic tools. Considering that both UPS and ALP dysfunction have been linked to Parkinson’s disease pathology, one can envision the possibility of deploying LYTACs in the former and PROTACs in the latter case to prevent α-syn aggregation and disease progression^8,9^. For example, as some forms of Parkinson’s disease are associated with mutations in lysosomal genes, PROTACs can be used to modulate α-syn levels as demonstrated by Qu et al.^4^. However, in other forms of Parkinson’s disease, in which lysosomal function is intact, LYTACs could target extracellular α-syn oligomers, preventing cell-to-cell transmission of α-syn. The availability of a variety of antibodies against different α-syn species that bind either or both monomeric and fibrillar α-syn raises exciting prospects for the development of novel LYTACs^10^. In conclusion, these two types of protein degradation systems hold tremendous promise for personalized medicine that can be customized based on the genetic background of the patient.

## References

[1] Crews CM (2010). Targeting the undruggable proteome: the small molecules of my dreams. Chem. Biol..

[2] Wu T (2020). Targeted protein degradation as a powerful research tool in basic biology and drug target discovery. Nat. Struct. Mol. Biol..

[3] Mullard A (2020). Targeted degraders clear first safety hurdles. Nat. Rev. Drug Discov..

[4] Qu J (2020). Specific knockdown of alpha-synuclein by peptide-directed proteasome degradation rescued its associated neurotoxicity. Cell Chem. Biol..

[5] Banik SM (2020). Lysosome-targeting chimaeras for degradation of extracellular proteins. Nature.

[6] Ahn, G. et al. Lysosome targeting chimeras (LYTACs) that engage a liver-specific asialoglycoprotein receptor for targeted protein degradation. 10.26434/chemrxiv.12736778.v1 (2020).

[7] Nakagawa T (2000). A novel motor, KIF13A, transports mannose-6-phosphate receptor to plasma membrane through direct interaction with AP-1 complex. Cell.

[8] Smolders S, Van Broeckhoven C (2020). Genetic perspective on the synergistic connection between vesicular transport, lysosomal and mitochondrial pathways associated with Parkinson’s disease pathogenesis. Acta Neuropathol. Commun..

[9] Ebrahimi-Fakhari D, Wahlster L, McLean PJ (2012). Protein degradation pathways in Parkinson’s disease - curse or blessing. Acta Neuropathol..

[10] Fields CR, Bengoa-Vergniory N, Wade-Martins R (2019). Targeting alpha-synuclein as a therapy for Parkinson’s disease. Front. Mol. Neurosci..

